# Supplementary material for: Venomix: a simple bioinformatic pipeline for identifying and characterizing toxin gene candidates from transcriptomic data
Source: PeerJ. 2018 Jul 31;6:e5361. doi: 10.7717/peerj.5361 (PMC6074769; doi:10.7717/peerj.5361)
Supplement: Supplemental Information 6 [file peerj-06-5361-s006.gz › FinalOutput_1E-6/Disintegrin-like_leberagin-C_8/finaltree.pdf]

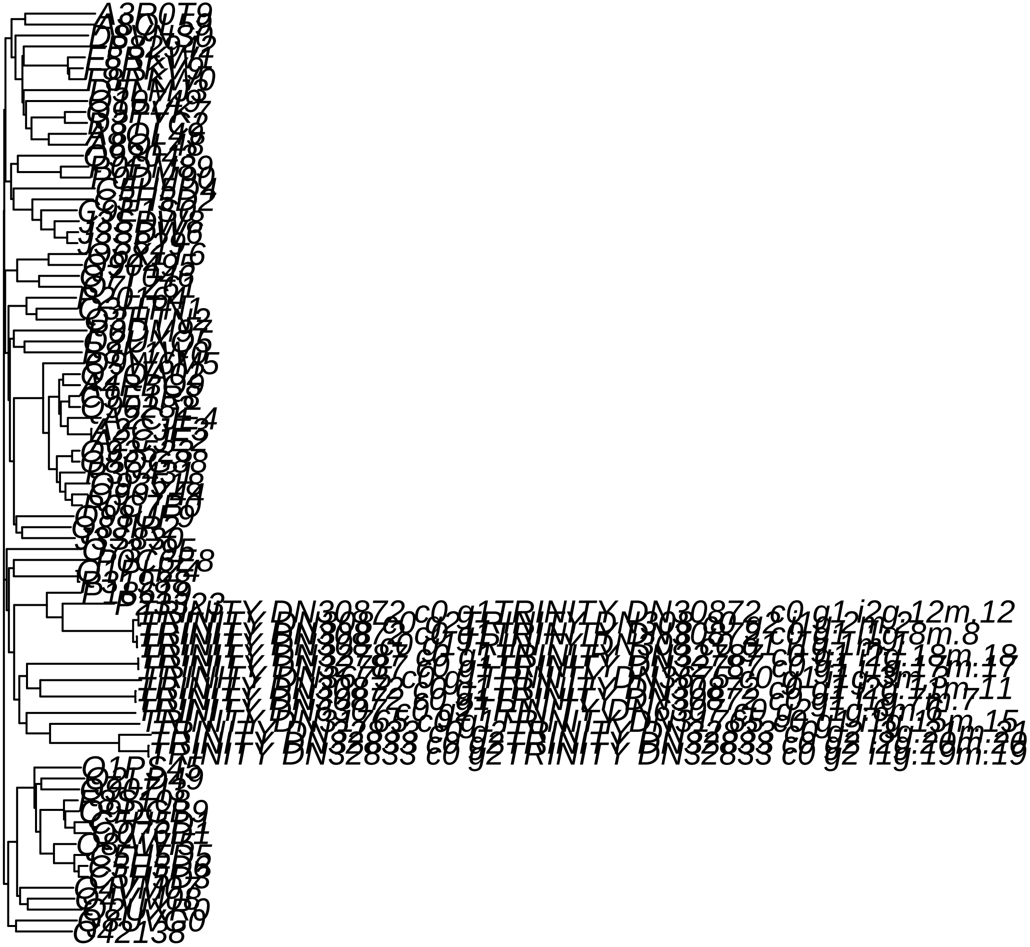

A3R0T9
TRINITY DN30872 c0 g1TRINITY DN30872 c0 g1 i2g.12m.12
TRINITY DN32833 c0 g2TRINITY DN32833 c0 g2 i1g.19m.19
Q42138
